# Uncovering Nematicidal Natural Products from *Xenorhabdus* Bacteria

**DOI:** 10.1021/acs.jafc.1c05454

**Published:** 2022-01-04

**Authors:** Desalegne Abebew, Fatemeh S. Sayedain, Edna Bode, Helge B. Bode

**Affiliations:** † Molekulare Biotechnologie, Goethe Universität Frankfurt, Max-von-Laue-Str. 9, Frankfurt am Main 60438, Germany; ‡ Department of Natural Products in Organismic Interactions, Max-Planck-Institute for Terrestrial Microbiology, Marburg 35043, Germany; § Senckenberg Gesellschaft für Naturforschung, Frankfurt am Main 60325, Germany

**Keywords:** entomopathogenic bacteria, *Xenorhabdus*, nematicidal natural products, cell-free culture supernatants, *Caenorhabditis elegans*, *Meloidogyne javanica*

## Abstract

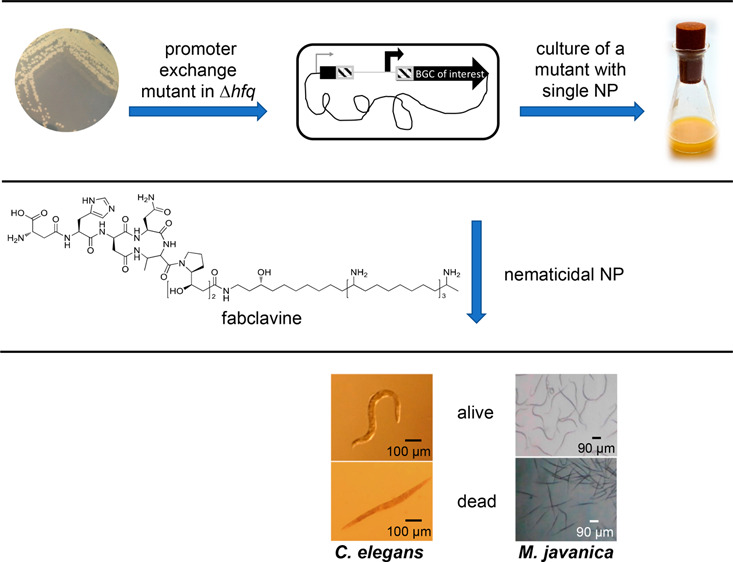

Parasitic nematodes infect different species of animals and plants. Root-knot nematodes are members of the genus *Meloidogyne*, which is distributed worldwide and parasitizes numerous plants, including vegetables, fruits, and crops. To reduce the global burden of nematode infections, only a few chemical therapeutic classes are currently available. The majority of nematicides are prohibited due to their harmful effects on the environment and public health. This study was intended to identify new nematicidal natural products (NPs) from the bacterial genus *Xenorhabdus*, which exists in symbiosis with *Steinernema* nematodes. Cell-free culture supernatants of *Xenorhabdus* bacteria were used for nematicidal bioassay, and high mortality rates for *Caenorhabditis elegans* and *Meloidogyne javanica* were observed. Promoter exchange mutants of biosynthetic gene clusters encoding nonribosomal peptide synthetases (NRPS) or NRPS-polyketide synthase hybrids in *Xenorhabdus* bacteria carrying additionally a *hfq* deletion produce a single NP class, which have been tested for their bioactivity. Among the NPs tested, fabclavines, rhabdopeptides, and xenocoumacins were highly toxic to nematodes and resulted in mortalities of 95.3, 74.6, and 72.6% to *C. elegans* and 82.0, 90.0, and 85.3% to *M. javanica*, respectively. The findings of such nematicidal NPs can provide templates for uncovering effective and environmentally safe alternatives to commercially available nematicides.

## Introduction

Parasitic nematodes infect many species of plants and animals, including humans causing serious diseases that are deleterious to human health and agricultural productivity.^1^ Nematodes that parasitize plants are a global problem for agriculture.^2,3^ Plant parasitic nematodes represent a significant constraint on global food security. Worldwide, they account for a loss of important agricultural crops, estimated to be about multiple billions of dollars per year.^4^*Meloidogyne incognita*, *M. javanica*, and *M. arenaria* are the most pathogenic *Meloidogyne* species. Root-knot nematodes are obligate plant parasites that exist in the roots of plants and interact with other plant pathogens to form disease complexes.^5^ Mostly root-knot nematodes affect development of host cells and gene expression and create giant cells, which affect absorption of water and nutrients from the soil.^6^

Administration of anthelmintic drugs (e.g., albendazole; ivermectin) is the major means of controlling human and animal nematode infections. However, many anthelmintic drugs are losing their effectiveness because nematode strains with resistance are emerging.^7,8^ Different strategies, such as using chemical pesticides, organic fertilizers, resistant host plants, and biological control, have been reported to control root-knot nematodes.^9^ However, application of chemical pesticides against nematode pests (e.g., methyl bromide) has declined due to high concerns for environmental welfare and increased demands of organic agriculture.^10,11^

Such problems stress the discovery of new and environmentally friendly nematicides. Soil bacteria can be a source of different biologically active compounds of economic and clinical importance.^12^ The genera *Xenorhabdus* and *Photorhabdus* are insect pathogenic bacteria, which exist in symbiosis with *Steinernema* and *Heterorhabditis* nematodes, respectively.^13^ After infection, these entomopathogenic bacteria produce a variety of bioactive compounds and hence kill the host insect larvae. These compounds protect the insect cadaver against food competitors, including bacteria and fungi.^14^

Since other nematodes living in the soil are also food competitors, entomopathogenic bacteria are expected to be potential sources of lead molecules for nematicidal chemicals. Hence, we hypothesized that some species of *Xenorhabdus* produce active compound(s) in their NPs that can be toxic to plant parasitic nematodes. Production of specific NPs was achieved by generating Δ*hfq* mutants of the desired strains that have a reduced background of NP production followed by activation of individual biosynthetic gene clusters via a promoter exchange mutant strategy.^18^ Accordingly, we tested cell-free culture supernatant*s* of different strains of *Xenorhabdus* for their nematode toxicity. Preliminary work in our laboratory showed that cell-free supernatants of some entomopathogenic bacteria killed second stage juvenile (J2) of *M. javanica* and prevented the egg hatching of this nematode.^15^ In this study, the nematicidal phenotypes of *Xenorhabdus* bacteria were characterized, and potent nematicidal NPs were identified, which were exclusively produced through engineering of their corresponding nonribosomal peptide synthetases (NRPS) or NRPS-polyketide synthase (PKS) hybrids encoding biosynthetic gene clusters.

## Materials and Methods

### Culturing Bacterial Strains

*Xenorhabdus* bacteria were isolated from their symbiotic nematode species (Table S1), which infected insect larvae, *Galleria mellonella*.^16^ Bacterial strains were stored in glycerol suspensions (50% v/v) at −80 °C and were cultivated on Luria-Bertani (LB) agar plates (15 g/L agar). *Xenorhabdus* and *Escherichia coli* strains were grown overnight with shaking at 30 and 37 °C, respectively, in LB broth (10 g/L tryptone, 5 g/L yeast extract, and 5 g/L NaCl at pH 7.0). Cultures were subsequently inoculated (1:100 v/v) in fresh LB media and incubated with a rotary shaker for 2 days. The liquid culture was supplemented, when it is required, with 0.2% l-arabinose and antibiotics in appropriate concentrations (ampicillin 100 μg/mL; kanamycin 50 μg/mL).^17,18^

### Cultivation of *C. elegans*

Culturing of wild type (WT) Bristol N2 strain of *C. elegans* was performed under standard culturing conditions on a nematode growth medium (NGM) agar plate (3 g/L NaCl, 2.5 g/L peptone, and 17 g/L agar). After autoclaving, the following ingredients were added as sterile filtered solutions: 1 mL of 1 M CaCl_2_, 1 mL of 1 M MgSO_4_, 25 mL of 1 M K_3_PO_4_, and 1 mL of cholesterol (5 mg/mL in EtOH). *C. elegans* is usually grown in the laboratory using *E. coli* OP50 strain as a food source. Overnight culture of *E. coli* OP50 (100 μL per plate) was spread on the NGM agar plate and grown at 37 °C for 12 h. *C. elegans* was transferred from one plate to another using a worm picker or a sterilized scalpel or spatula, and it was cultivated on the bacterial lawn for 3 days at 25 °C.^19^

### Cultivation of *M. javanica*

A culture of plant parasitic nematode *M. javanica* was maintained on the tomato plants in the greenhouse for 3 months. Tomato roots with egg masses were washed, and hand-picked egg sacs of *M. javanica* were placed on a nylon screen immersed in shallow water in glass Petri dishes, and hatched second stage juveniles (J2) were disinfected daily with streptomycin sulfate (0.1%) for 15 min and then were washed with distilled water to be used for the nematicidal bioassay.^20^

### DNA Techniques and DNA Manipulation

Techniques for plasmid DNA preparation, restriction digestion, transformation, and DNA gel electrophoresis were adapted from standard protocols.^21^ Isolation of genomic DNA was carried out based on the manufacturer’s instructions (QIAGEN). PCR amplifications were carried out on thermocyclers (SensoQuest). Restriction enzymes and DNA polymerases (Taq, Q5, and Phusion) were purchased from New England Biolabs or Thermo Fisher Scientific. DNA primers were purchased from Eurofins MWG Operon. The general plasmids used in this work are listed in Table S2. The PCR primers used in this study are shown in Table S4. All plasmids generated in this study (Table S4) were constructed using Hot Fusion Cloning.^22^

### Construction of Deletion Mutants

Deletion mutants in *X. doucetiae* DSM 17909 and *X. budapestensis* DSM 16342 were created using the primers listed in Table S4. The *hfq* gene of these two strains was deleted to abolish or reduce production of NPs.^18^ The *hfq* gene was deleted by amplifying about 1 kb fragments upstream and downstream of the respective genes. The amplified fragments were cloned into the either digested or PCR-amplified pCK_cipB backbone by Hot fusion assembly and then transformed into *E. coli* S17-1 λpir. Conjugation of the plasmid in *Xenorhabdus* and the generation of double crossover mutants via counter selection were done following established protocols.^22^ Verification of deletion clones was confirmed via PCR with the verification primers listed in Table S4.

### Generation of Promoter Exchange Mutants

Promoter exchange mutants in *Xenorhabdus* were created using the primers listed in Table S4.^17,18^ These strains were generated for the production of specific NPs using the exchange of the natural promoter against an arabinose-inducible P_BAD_ promoter. Promoter exchange mutants of *Xenorhabdus* were constructed following standard protocols.^17^ Briefly, the pCEP plasmid carrying the first 600–800 bp of a gene of interest of *Xenorhabdus* was constructed by Hot Phusion and transformed into *E. coli* S17-1 λpir. Cell suspensions of transformed *E. coli* were plated on selected LB agar plates containing kanamycin 50 μg/mL. Verification of positive clones was carried out via colony PCR using verification primers (Table S3). *Xenorhabdus* strains were conjugated with positive clones of *E. coli* S17-1 λpir harboring the respective promoter exchange plasmid as indicated previously. For both strains, overnight cultures were prepared in the LB liquid medium using appropriate antibiotics (kanamycin 50 μg/mL and ampicillin 100 μg/mL). The next day, both strains were grown in 5 mL of LB medium to an OD_600_ of 0.6–0.8. The cells were harvested using 1 mL from each strain and washed using a fresh LB medium. The cells of *E. coli* S17-1 λpir were resuspended in 100 μL of LB, and *Xenorhabdus* bacteria were mixed on the LB agar plate in a ratio of 1:3 and incubated at 37 °C for 3 h and transferred to 30 °C until the next day. After 1 day, the cells were resuspended in 2 mL of LB for plating 100 μL of cell suspension on selective LB agar plates supplemented with kanamycin 50 μg/mL and ampicillin 100 μg/mL for further antibiotic resistance selection. The cells were incubated at 37 °C for 72 h. Screening of clones was carried out genetically by PCR using verification primers (Tables S3 and S4).^17^

### Fermentation and Cell-Free Culture Supernatant Preparation

*Xenorhabdus* bacteria listed in Table S1 were used to harvest cell-free culture supernatants for testing their nematicidal activity. They were cultured for 2 days at 200 rpm on a rotary shaker in LB broth at 30 °C. The cultures were cultivated in 1 L Erlenmeyer flasks containing 100 mL + 0.2% l-arabinose and inoculated with a 24 h preculture (0.1%, v/v). For the preculture, appropriate antibiotics were added to the LB medium when necessary at the following concentrations: kanamycin 50 μg/mL and ampicillin 100 μg/mL. The cell-free culture supernatants were prepared by centrifugation at 4000 rpm for 30 min in 50 mL Falcon tubes and filtered through a 0.2 μm filter. The supernatants were heat-treated at 90 °C for 10 min so that protein toxins denature and their effect can be separated from the NPs. Culture supernatants of *E. coli* OP50 and *Xenorhabdus* strains (WT and Δ*hfq*) were used as controls. Additionally, freeze-dried supernatants of bacterial strains were prepared. Production of specific NPs from each promoter exchange strain was analyzed using HPLC-MS or MALDI-MS before being used for nematicidal bioactivity testing. For HPLC-MS analysis, strains were cultured in 5 mL of LB liquid medium with 0.2% l-arabinose and 2% Amberlite XAD-16 resin. After 72 h, XAD-16 beads were separated and extracted with 5 mL of MeOH for 1 h. The cell-free supernatant of the strains was used for MALDI-MS analysis.

### Nematicidal Activity Test against *C. elegans*

Solid assay was adapted from the plate assay described by Tan et al.^23^ NGM agar plates (35 mm in diameter) were seeded with an overnight culture of bacteria (100 μL). The plates were incubated at 30 °C (*Xenorhabdus* strain) and 37 °C (*E. coli* OP50) for 48 and 24 h, respectively, to enable growth of the bacteria. L4 stages of *C. elegans* (up to 50 larvae per plate) were added onto each NGM. Lids of the plates were covered with parafilm. Incubation of plates was done at 25 °C, and death of the nematodes was analyzed every 24 h. Worms were considered dead after being unresponsive upon tapping the plate under a microscope.

The nematicidal activity against *C. elegans* was determined in a 24-well microtiter plate by a slightly modified method,^24−26^ where the cell-free culture supernatant of different bacterial strains was added for testing. Nematodes grown on the NGM seeded with an *E. coli* OP50 lawn of cells were washed from the plates with M9 buffer (3 g of KH_2_PO_4_, 6 g of Na_2_HPO_4_, 5 g of NaCl, and, after autoclaving, the addition of 1 mL of 1 M MgSO_4_). Finally, a nematode suspension was filtered through a sieve with pores of 40 μm. In this assay, 80–100 L4-stage *C. elegans* were added in to a well of the 24-well microtiter plate containing 300 μL of cell-free culture supernatant from a bacterial strain to be tested. The plates were incubated at 25 °C in the dark, and the viability of the worms was recorded under a stereomicroscope at 40× magnification every 24 h for 3 days. The cell-free culture supernatant of *E. coli* OP50 was used as a negative control. The killing assay was conducted in triplicate. The nematodes were classified as dead when no movement was observed under a stereomicroscope and when their bodies were straightened. Mortality of the nematodes was calculated as the ratio of dead nematodes compared to the total number of tested nematodes.

### Nematicidal Activity Test against *M. javanica*

To evaluate the nematicidal effect on second stage juveniles (J2) of root-knot nematodes (*M. javanica*), 1.5 mL of sterile distilled water was added to each 15 mL Falcon tube containing the freeze-dried supernatant, resulting in a 10-fold concentration of the original supernatant. Then, the solutions were filtered through the 0.2 μm filter and were tested against J2 *M. javanica*. About 100 J2s of root-knot nematodes were added in each well of a 24-well plate containing 0.50 mL of fresh supernatant of bacterial strains (0.45 mL of supernatant +0.03 mL of nematode suspension +0.02 mL of streptomycin sulfate 0.1%). The number of dead J2 was recorded after 24 and 48 h with the stereomicroscope. Juveniles without movement were considered dead, and they were touched with a fine needle to confirm their death. The experiment was conducted based on a completely randomized design with three replications.^20^

### Microscopy

Stereomicroscopy was used for counting live and dead worms during the nematode killing assay through a magnification of 40×. Detection of green fluorescent protein (GFP)-labeled *Xenorhabdus* in the gut of *C. elegans* was conducted using fluorescent microscopy. *C. elegans* was stained with Nile-Red stain as the control following an established protocol.^27^

### Statistical Analysis

Analysis of variance (ANOVA) for all the obtained data was performed using the SAS (v. 9.1) software. Furthermore, the LSD test was employed for significant differences among treatments at *P* < 0.05.^28^

## Results and Discussion

### Nematicidal Activity of Lawn of Cells of *Xenorhabdus* Bacteria against *C. elegans*

**Figure 1:**
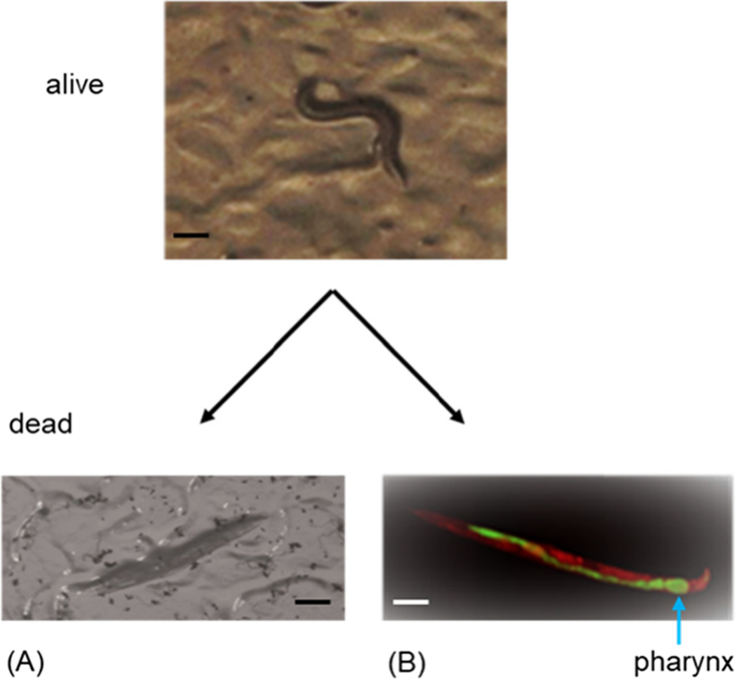
Nematicidal effect of *Xenorhabdus* bacteria on the L4 stage of *C. elegans*. (A) Nematodes transferred onto a lawn of *X. budapestensis* on NGM were killed within 2–3 days. Pictures were taken using a stereomicroscope. The scale bars are 100 μm. (B) GFP-labeled *X. nematophila* bacteria (green) were grazed from the NGM agar plate by *C. elegans* and found disseminated through the gut of the nematode, and the bacteria caused engorgement at the pharynx of the nematode. The nematodes were stained with Nile-Red stain (red) as a contrast, and pictures were taken using fluorescence microscopy. The scale bar is100 μm.

*C. elegans* is a free-living nematode, which typically grows on NGM agar plates containing *E. coli* OP50, which represents the standard laboratory food for *C. elegans*.^19,29^ Based on this information, *C. elegans* was grown on the lawn of cells of *X. budapestensis*, *X. szentirmaii*, *X. doucetiae*, and *X. nematophila*. As a result, we observed that the lawn of cells of *Xenorhabdus* bacteria killed *C. elegans* while grazing on it (Figure 1A). This initiated us to identify the cause of death of this free-living nematode. To verify whether *C. elegans* grazes *Xenorhabdus* bacteria into its intestine, we used GFP-labeled *Xenorhabdus* bacteria as a lawn of cells. In this experiment, we noticed that the GFP-labeled *Xenorhabdus* bacteria caused infection and distributed throughout the entire length of the nematode intestine. A mass of cells of the bacteria caused engorgement at the pharynx, near the mouth of *C. elegans* (Figure 1B). Most of *C. elegans* were found dead within 2–3 days of the experiment. Hence, *Xenorhabdus* bacteria either affect normal physiological function of the intestine of the nematodes or produce nematicidal NPs in the gut of the nematodes to kill them within a short period of time. Other studies reported that *C. elegans* was antagonized through the colonization of the intestine by different human pathogens such as *Salmonella typhimurium*(30) and *Pseudomonas aeruginosa*.^31^ Similarly, we showed that GFP-labeled *X. nematophila* bacteria disseminated through the intestine of *C. elegans*, which resulted in death of the worms over the span of a few days, although the precise mechanism of killing remained unknown.

### Nematicidal Activity of Cell-Free Culture Supernatants of *Xenorhabdus* Bacteria against *C. elegans*

Most of *Xenorhabdus* bacteria showed strong nematicidal activity against the L4 stage of *C. elegans* during the microtiter plate nematicidal assay. Cell-free culture supernatants of WT of *X. budapestensis*, *X. szentirmaii*, and *X. doucetiae* grown in the LB medium resulted in mortalities of 91.0, 90.3, and 77.0% for *C. elegans*, respectively, after 48 h of the experiment (Figure S1; Table 1). HPLC-MS data analysis was conducted for the cell-free culture supernatant of nematicidal *Xenorhabdus* bacteria (e.g., *X. budapestensis* WT) and non-nematicidal *E. coli* OP50 (control). The profile of their base peak chromatograms (Figure S3) agrees with their nematicidal activities (Figure S2). Even if there was no significant variation among them (*P* > 0.05), cell-free supernatants of WT of *X. budapestensis* and *X. szentirmaii* had the greatest nematicidal effect followed by *X. doucetiae* (Table 1). Our findings agree with earlier results that have shown that cell-free culture supernatants of *Xenorhabdus* and *Photorhabdus* possess nematicidal activity against nematodes.^15,32−37^

**Table 1:**
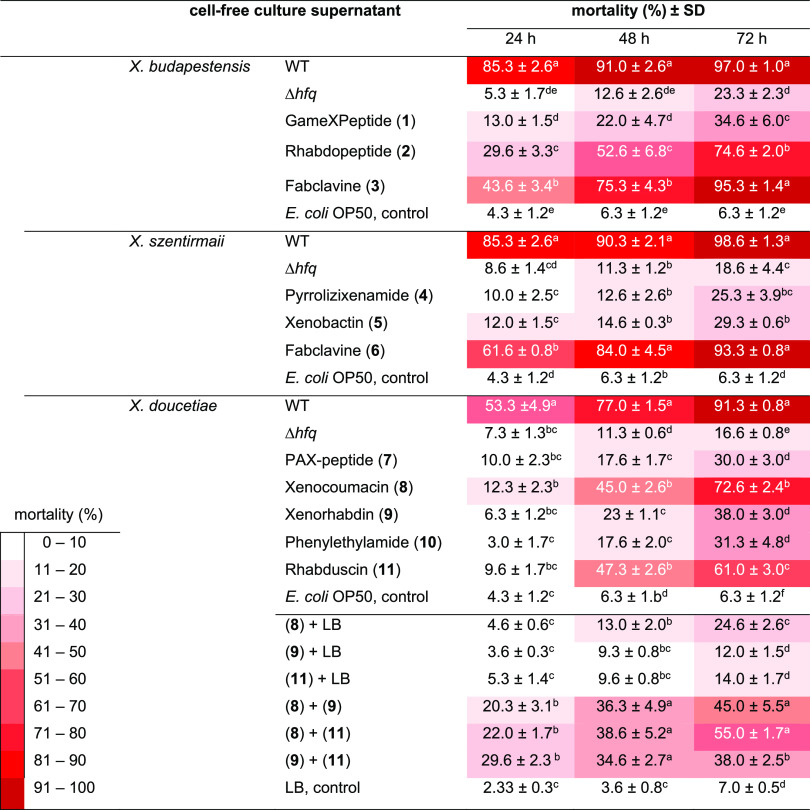
Nematicidal Activity of Cell-Free Culture Supernatants of *Xenorhabdus* Bacteria against the L4 Stage of *C. elegans*^a^

a Mean values represent the mean of triplicates. Means in each column indicated by the same letter are not significantly different at *P* < 0.05 according to the LSD test. Comparison of the mean values is conducted for each strain separately using their mean values at each day of the experiment. Supernatants of WT, their corresponding Δ*hfq* strains, and promoter exchange strains (Δ*hfq*-pCEP-NP; induced with 0.2% arabinose) were used. Cell-free culture supernatants of *E. coli* OP50 and LB liquid media were used as the control. The experiments were conducted in triplicate, and mean values of the mortality are indicated here. Bioactivities are shown for none (white) to the highest activity (red).

### Characterization of Nematicidal Phenotypes of *Xenorhabdus* Bacteria

**Figure 2:**
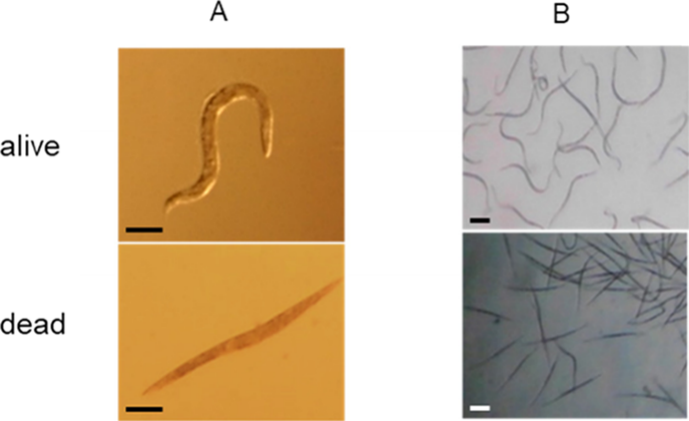
Microscopy of nematodes during the nematicidal bioactivity test. (A) Cell-free culture supernatant of *Xenorhabdus* kills *C. elegans* during the microtiter plate assay. The L4 stages of *C. elegans* were added into a 24-well microtiter plate containing 300 μL of cell-free culture supernatant of *Xenorhabdus* strains. The scale bars are 100 μm. (B) Cell-free culture supernatant of *Xenorhabdus* bacteria showed nematicidal activity against the root-knot nematode, second stage juveniles (J2) *M. javanica*. The scale bars are 90 μm.

**Figure 3:**
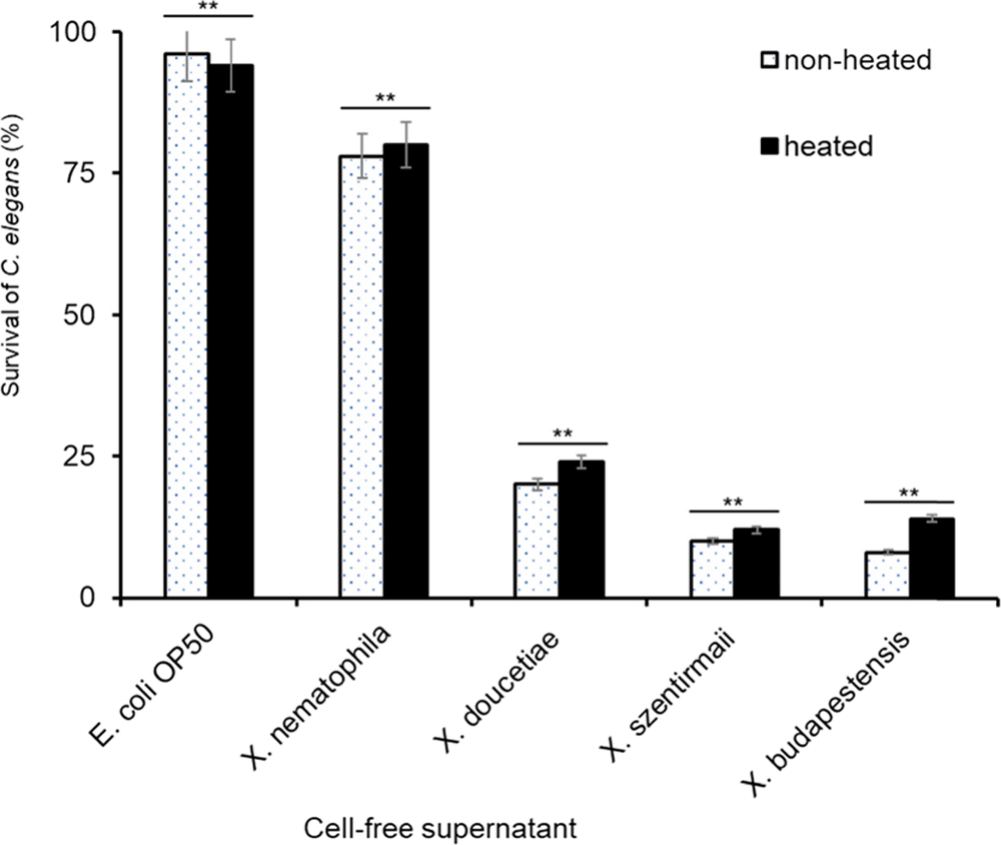
Effect of heat inactivation of the cell-free culture supernatant on nematicidal activity. Heating of the cell-free culture supernatant does not affect the nematicidal activity of different WT of *Xenorhabdus* bacteria against *C. elegans* (L4). Supernatants were heated at 90 °C for 10 min to inactivate protein toxins. The survival rate of *C. elegans* was calculated after 48 h of the experiment. Heated cell-free culture supernatants have nearly the same nematicidal activity as the non-heated ones; this indicates the presence of nematode toxic NPs in heated cell-free culture supernatants. ** indicates the absence of significant difference (*P* > 0.05) between the nematicidal activity of heated and nonheated supernatants.

We showed that cell-free culture supernatants of WT of *Xenorhabdus* bacteria killed *C. elegans* (Table 1, Figure 2A, and Figure S1), which could be due to either protein toxins or NPs. To differentiate the real cause of death for the nematode, cell-free culture supernatants were heated to inactivate protein toxins in cell-free culture supernatants of *X. szentirmaii*, *X. budapestensis*, *X. nematophila*, and *X. doucetiae*. All supernatants kept a high nematicidal activity after heat treatment at 90 °C for 10 min (Figure 3). This result indicated that the nematicidal compounds produced by these bacteria are heat stable as suggested for small molecule NPs and unlike toxic proteins. Even if toxic proteins also have a similar effect,^38^ our results demonstrated that *Xenorhabdus* are capable of fast killing *C. elegans* through production of NPs in their culture supernatants.

The gene encoding Hfq has been shown to influence virulence in some pathogens like *Pseudomonas aeruginosa* and *Salmonella typhimurium* and production of NPs in *Photorhabdus* and *Xenorhabdus* bacteria.^39,40^ After Δ*hfq* mutant strains of *X. szentirmaii*, *X. budapestensis*, and *X. doucetiae* were generated, we compared cell-free culture supernatants of Δ*hfq* strains with their corresponding WT for their nematicidal activity (Table 1). Cell-free culture supernatants of WT of *Xenorhabdus* bacteria show high nematode toxicity against *C. elegans*. In contrast, their corresponding Δ*hfq* strains did not show such toxicity and differed significantly (*P* < 0.05) (Table 1). The absence of nematicidal properties compared to the WT is related to deletion of the *hfq* gene, which is a global regulator of gene expression (production of NPs) through sRNA/mRNA interactions.^41^

### Identification and Exclusive Production of Nematicidal Natural Products from *Xenorhabdus* Bacteria

**Figure 4:**
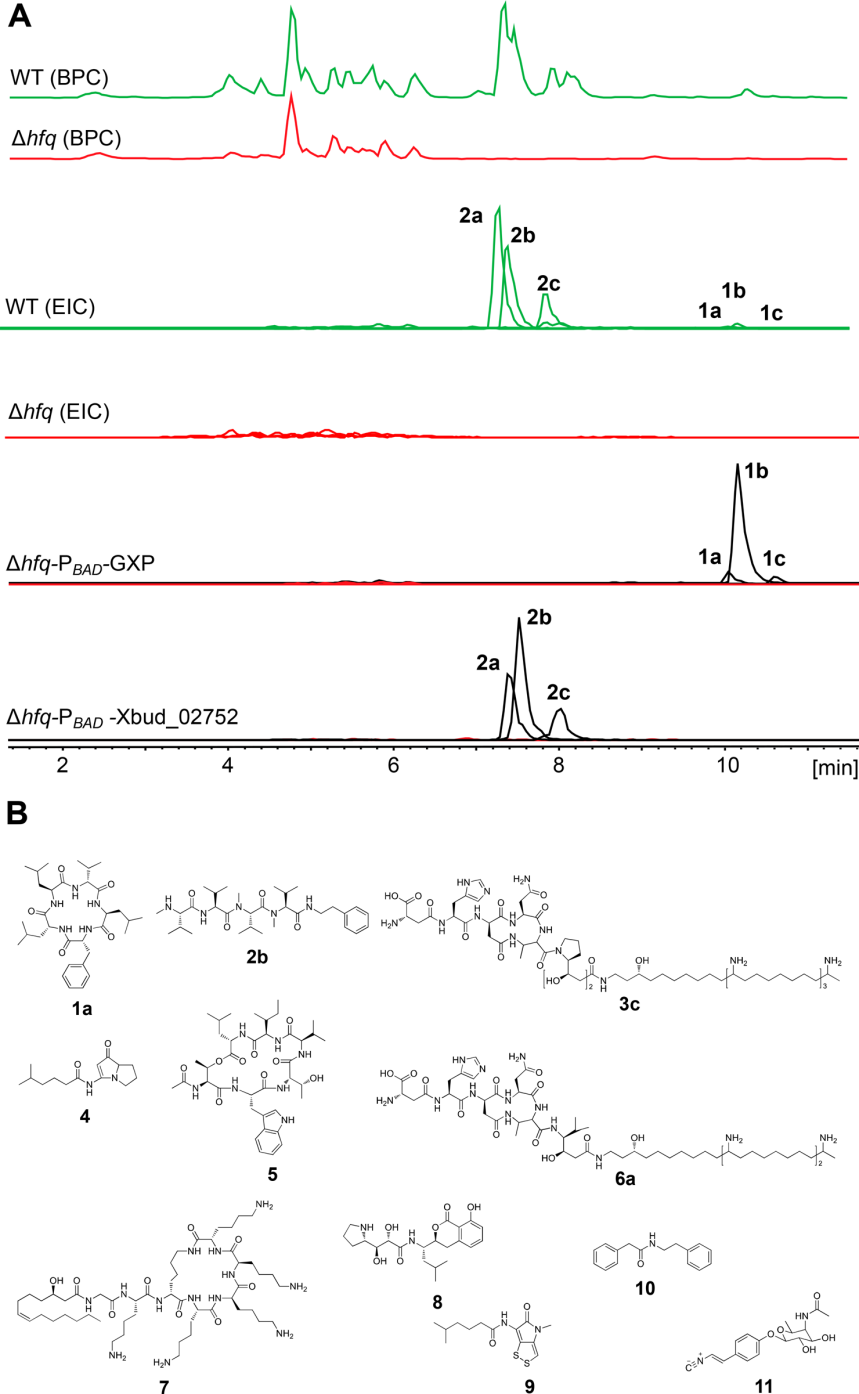
Exclusive production of natural products using promoter exchange via the easyPACId approach. (A) HPLC-MS data analysis of WT and Δ*hfq* mutants of *X. budapestensis* and NPs detected after a promoter exchange in the Δ*hfq* mutant. Extracted ion chromatograms (EICs) of derivatives of GameXPeptide (GXP) (**1a**, **1b**, and **1c**) and rhabdopeptide (Xbud_02752) (**2a**, **2b**, and **2c**) are indicated to show their exclusive production. (B) Structures of all NPs involved in the study.

We observed potent nematicidal activity from cell-free culture supernatants of WT of different *Xenorhabdus* bacteria. On the other hand, there was almost no nematicidal activity from their corresponding Δ*hfq* mutants (Table 1). From these two different phenotypes of a strain, it is possible to suggest that the nematicidal NP is produced by *hfq* dependent NRPS and NRPS-PKS hybrid biosynthetic gene clusters (BGCs). We first generated *hfq* mutant strains, which did not produce any NP known from the WT strains. Next, exclusive production of an NP was achieved using the easyPACId^18^ (easy Promoter Activation for Compound Identification) in which the native promoter was exchanged against an arabinose-inducible P_BAD_ promoter in the *hfq* mutant strain (Figures 4A and S4–S6).

Accordingly, we generated 11 different easyPACId strains for which nematicidal bioactivity was analyzed using the cell-free culture supernatants against the L4 stage of *C. elegans*, including controls of LB, *E. coli* OP50, Δ*hfq*, and the corresponding WT (Table 1). In *X. budapestensis*–Δ*hfq*, GameXPeptides (**1**), rhabdopeptides (**2**), and fabclavines (**3**) were exclusively produced using activation of the P_BAD_ promoter.^18^ Similarly, in *X. szentirmaii*–Δ*hfq*, pyrrolizixenamide (**4**), xenobactin (**5**), and fabclavines (**6**) were individually produced. PAX-peptide (**7**), xenocoumacin (XCN2) (**8**), xenorhabdin (**9**), phenylethylamide (**10**), and rhabduscin (**11**) were also produced from *X. doucetiae*–Δ*hfq* following the same procedure. The production status of these NPs was verified using HPLC-MS and/or MALDI-MS data analysis (Figure 4A; Figures S4–S6). We observed that activation of some BGCs could also result in production of an NP class having multiple derivatives (Table S5).

Out of 11 NPs that we tested, almost all resulted in more than 50% mortality of *C. elegans*. However, fabclavines (**3**; **6**), rhabdopeptides (**2**), and xenocoumacin (**8**) appeared to be the most nematicidal NPs showing mortality greater than 70% with fabclavines (**3**) in *X. budapestensis* strain being the most toxic with 95.3% mortality against *C. elegans* (Table 1).

Previously, fabclavines were described as antibacterial and antifungal with broad spectrum properties,^42,43^ and recent studies confirmed that fabclavines have potent feeding-deterrent activity against deadly mosquito vectors.^44^ Fabclavines also displayed antibacterial efficacy against a multidrug-resistant *E. faecalis*.^45^ Additionally, it was demonstrated that zeamine, identified in *Serratia plymuthica*,^46^ and fabclavines^42^ show similarity in their structures containing a polyamine moiety. It was hypothesized that zeamine cytotoxicity involves disruption of the plasma membrane to facilitate solubilization of nematode cuticles.^12^ Hence, fabclavines might have a similar mechanism of action, but this is a subject for future in-depth investigation. However, most of nematicidal drug classes impair the neuromuscular system of nematodes by interacting with ion channels and receptors on neurons and muscles.^47^

It was reported that rhabdopeptides contribute to insect killing acting as insect specific virulence factors^48^ and displayed positive effect against protozoal parasites.^49^ In our work, we observed nematicidal activity of rhabdopeptides against *C. elegans*.

### Toxicity of Cell-Free Culture Supernatants of *Xenorhabdus* Bacteria against *M. javanica*

The most active nematicidal compounds that we identified were also tested for their activity against root-knot nematodes (Figure 2B). Although nematicidal activity was shown in all of fresh supernatants of bacterial mutants, only freeze-dried supernatants of fabclavines (**6**), rhabdopeptides (**2**), and xenocoumacin (**8**) resulted in 82.0, 90.0, and 85.3% mortalities of J2 of *M. javanica*, respectively, after 48 h (Table 2). A study reported that ammonium produced by *Xenorhabdus* causes nematicidal activity on J2 of *M. incognita*.^33^ Indole and 3,5-dihydroxy-4-isopropylstilbene (IPS), from the culture filtrate of *P. luminescens* MD, were shown to have nematicidal activity. IPS caused mortality of *C. elegans* but had no effect on J2 of *M. incognita*, while indole was lethal to *M. incognita*.^34^ Extracts of *P. luminescens* CH35 showed nematicidal activity on *M. incognita* but, however, had weak effect on *C. elegans*.^50^ Hence, entomopathogenic bacteria have great potential to produce different nematicidal NPs.

**Table 2:**
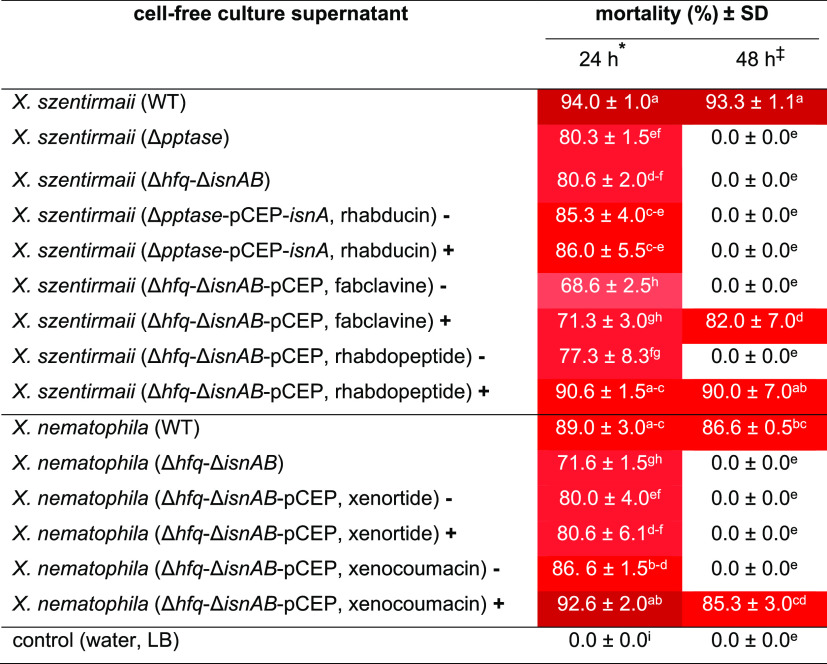
Nematicidal Effect of *Xenorhabdus* Bacteria against *M. javanica* (J2)^a^

a Mean values in each column indicated by the same letter are not significantly different at *P* < 0.05 according to the LSD test. + represents induced natural product in strain. - represents not induced natural product in strain. * represents mean of mortality in fresh supernatants. ‡ represents mean of mortality in freeze-dried supernatants. Fresh and freeze-dried cell-free supernatants of WT and their corresponding promoter exchange strains (Δ*hfq*-pCEP-NP; induced with 0.2% arabinose) were used. Sterile water and LB liquid media were used as the control. The experiments were conducted in triplicate, and mean values of the mortality are indicated here. Bioactivities are shown for none (white) to the highest activity (red), see Table 1 for the color code.

Three types of rhabdopeptides from *X. budapestensis* SN84 have been recently isolated that indicated nematicidal properties on *M. incognita*.^51^ Our results were in agreement with these results since we observed a similar nematicidal activity of cell-free culture supernatants of rhabdopeptides **2** producing strains. During detailed HPLC-MS analysis of an induced promoter exchange strain of *X. budapestensis*–Δ*hfq*-P_BAD_-Xbud-02752, we detected different derivatives of rhabdopeptides **2**, which could be isolated and purified in the future to study their structure–activity relationship (Figure 4A).

In conclusion, *Xenorhabdus* bacteria produced a variety of nematicidal NPs. We identified the responsible NPs from the liquid culture of different strains of *Xenorhabdus* bacteria by applying the easyPACId approach of exclusive production of NPs, which was achieved through engineering of the corresponding NRPS and NRPS-PKS encoding BGCs. Herein, we enabled the strains to produce exclusively the active compounds. In addition, such microbially produced NPs are in principle degradable and ecofriendly for agricultural application. This makes them potentially useful for the biocontrol of nematodes in crop and vegetable production. We are still missing greenhouse data and toxicity data of the compounds against plants and other soil organisms including other insects. However, it is worth studying the mechanism of action of these promising nematicidal compounds in detail in the future. By conducting a close structural investigation of the promising NPs, it will be possible to design and synthesize the best and safest nematicidal drug from them. Therefore, our findings may create new avenues toward the development of efficient and safe nematicidal compounds that can be used to enhance the quality of animal and crop productions.

## Supporting Information Available

Tables of strains, plasmids, primers, and natural products; nematicidal activity of culture supernatants, HPLC/MS chromatograms, and MALDI-MS spectra of bacterial strains (PDF)

## Abbreviations

BGC: biosynthetic gene cluster
bp: base pairs
BPC: base peak chromatogram
DNA: deoxyribonucleic acid
EIC: extracted ion chromatogram
EtOH: ethanol
fcl: fabclavine
GFP: green fluorescent protein
Gxp: GameXPeptide
h: hour
Hfq: host factor of the RNA bacteriophage Qß
HPLC: high-performance liquid chromatography
IJ: infective juvenile
Kb: kilobase
LB: lysogeny broth
MALDI: matrix-assisted laser desorption/ionization
MeOH: methanol
min: minute
MS: mass spectrometry
*m*/*z*: ratio of mass to charge
NGM: nematode growth medium
NP: natural product
NRPS: nonribosomal peptide synthetase
OD_600_: optical density at a wavelength of 600 nm
P_BAD_: l-arabinose-inducible promoter
pCEP: cluster expression plasmid
PCR: polymerase chain reaction
PKS: polyketide synthase
PPTase: phosphopantetheine transferase
Rdp: rhabdopeptide
WT: wild type
XCN: xenocoumacin
