# Supplementary material for: Uncovering Nematicidal Natural Products from Xenorhabdus Bacteria
Source: J Agric Food Chem. 2022 Jan 4;70(2):498–506. doi: 10.1021/acs.jafc.1c05454 (PMC8778618; doi:10.1021/acs.jafc.1c05454)
Supplement: Supplementary file 1 — jf1c05454_si_001.pdf [file jf1c05454_si_001.pdf]

Supplementary Information

Uncovering nematicidal natural products from *Xenorhabdus* bacteria

Desalegne Abebew[1], Fatemeh S. Sayedain[2], Edna Bode[2] and Helge B. Bode[1,2,*]

[1] Molekulare Biotechnologie, Goethe Universität Frankfurt, Max-von-Laue-Str. 9, 60438 Frankfurt am Main, Germany
[2] Department of Natural Products in Organismic Interactions, Max-Planck-Institute for Terrestrial Microbiology, 35043, Marburg, Germany

* Corresponding author: Tel.: +49 6421 178 501, Fax: + 49 6421 178 509,
Email: helge.bode@mpi-marburg.mpg.de

Table of content

Table S1. WT of *Xenorhabdus bacteria* and nematode host species. 2

Table S2. List of general plasmids used in this work. 2

Table S3. List of general primers used in this work. 3

Table S4. List of primers and plasmids used to generate new *Xenorhabdus* strains.. 4

Table S5. NPs mentioned in this work including derivatives for some NPs. 6

Figure S1. Nematicidal activity of cell-free culture supernatant of *Xenorhabdus* WT strains 7

Figure S2. Nematicidal activity of cell-free culture supernatant of *X. budapestensis* WT compared to its Δ*hfq* strain 8

Figure S3. HPLC-MS data analysis for WT of *Xenorhabdus* and *E. coli* OP50. 9

Figure S4. Exclusive production of natural products using promoter exchange. 10

Figure S6. MALDI-MS spectra of fabclavine in *X. szentirmaii* WT and its promoter exchange mutant. 12

References.. 13

Table S1. WT of *Xenorhabdus bacteria* and nematode host species. Wild type strains used in nematicidal bioassay and nematode host species of *Xenorhabdus* bacteria.

Strain	Genotype	Reference	Nematode host
E. coli S17-1λpir	Tpr Smr recA thi rsdRM+ RP4::2-Tc::Mu::KM Tn7, λ pir phage lysogen	Invitrogen	-
E. coli OP50	wild type	[1]	-
C. elegans Bristol N2	wild type	[2]	-
M. javanica	wild type	[3]	-
X. doucetiae DSM 17909	wild type	[4]	*Steinernema diaprepesi*
X. nematophila HGB081	wild type	[5]	*Steinernema carpocapsae*
X. szentirmaii DSM 16338	wild type	[6]	*Steinernema rarum*
X. budapestensis DSM 16342	wild type	[7]	*Steinernema bicornutum*

Table S2. List of general plasmids used in this work.

Plasmid	Genotype	Reference
pCEP_kan	pDS132 based, R6K ori, oriT, kan^R, *araC*, P_{BAD}	[8]
pEB17	pDS132 based, R6K ori, oriT, kan^R, *cipB*, *sacB*	[9]

Table S3. List of general primers used in this work.

Oligonucleotide	Sequence (5´-3´)
VpDS132-fw	GATCGATCCTCTAGAGTCGACCT
VpDS132-rv	ACATGTGGAATTGTGAGCGG
VpCEP-fw	GCTATGCCATAGCATTTTTATCCATAAG
pCEP-BB-fw-gib	ATGTGCATGCTCGAGCTC
pCEP-BB-rv-gib	ATGCTAGCCTCCTGTTAGC

Table S4. List of primers and plasmids used to generate new *Xenorhabdus* mutant strains.

Strain	Oligo-nucleotide	Sequence (5´- 3´)	Plasmid in *E. coli* S17-1λpir	construction method	Natural product	Reference
***X. szentirmaii* DSM 16338**						
X. szentirmaii Δ*hfq*						[10]
X. szentirmaii Δ*hfq* pCEP-KM-3680					xenobactin	[10]
X. szentirmaii Δ*hfq* pCEP-KM-5118					pyrrolizixenamide	[10]
X. szentirmaii Δ*hfq* pCEP-KM-*fclC*					fabclavine	[10]
***X. nematophila* HGB081**						
X. nematophila Δ*hfq*						[10]
X. nematophila Δ*hfq* pCEP-xenortide					xenortide	[10]
X. nematophila Δ*hfq* pCEP-xenocoumacin	For the construction of these strains see details in the given references				xenocoumacin	[10]
***X.* doucetiae DSM 17909**						
X. doucetiae Δ*hfq*						[10]
X. doucetiae Δ*hfq* pCEP- phenylethylamide					phenylethylamide	[8]
X. doucetiae Δ*hfq* pCEP-rhabduscin					rhabduscin	[10]
X. doucetiae Δ*hfq* pCEP-PAX-peptide					PAX-peptide	[10]
X. doucetiae Δ*hfq* pCEP-xenocoumacin					xenocoumacin	[10]
X. doucetiae Δ*hfq* pCEP-xenorhabdin					xenorhabdin	[10]
***X. budapestensis* DSM 16342**						
DSM 16342Δ*hfq*	DAS_07_fw	TCGATCCTCTAGAGTCGACCTGCAGATCGCTTTACGTCAGCATCT	pCK_cipB_DSM16342Δ*hfq*			this study
	DAS_08_rv	TCAGCGATATCATTTTCCTGTTGTGCTGGATCTTGCAAAGATTG				
	DAS_09_fw	TAAGGGGCAATCTTTGCAAGATCCAGCACAACAGGAAAATGATA				

Strain	Oligo-nucleotide	Sequence (5´- 3´)	Plasmid in *E. coli* S17-1λpir	construction method	Natural product	Reference
	DAS_10_rv	GGAATTCCCGGGAGAGCTCAGA TCTGCATCAACAACATGAAGCAG				
	DAS_15-_VP_fw	GCTTATTCCATTGAAACTGT				
	DAS_ 16 _VP_rv	GAAAGACGCTCAGTCAGTG				
DSM 16342_Δ*hfq*_P_{BAD}_Xbud_ 22634_km	DAS_38_ fw	TTTGGGCTAACAGGAGGCTAGC ATATGAATTACCCTGAAACCT	pCEP_Xbud _22634_KM	[9]	fabclavine	this study
	DAS_39_ rv	TCTGCAGAGCTCGAGCATGCAC ATGGCTGTTATCATACTTAAGC				
	DAs_40_VP _rv	CATCAGCATTCTGATCGAAG				
DSM 16342_Δ*hfq*_P_{BAD}_Xbud_ 02752_km	DAS_119_ fw	TTTGGGCTAACAGGAGGCTAGC ATATGAAAAATGCAGCTAAAAT	pCEP_Xbud _02752_KM	[9]	rhabdopeptide	this study
	DAS_120_rv	TCTGCAGAGCTCGAGCATGCAC ATCCCCTTTATTATAAGCACG				
	DAS_121_V P_ rv	GTTGCTCATTAAGCTTCCCTTC				
DSM 16342_Δ*hfq*_P_{BAD}_ Xbud_01497_km	DAS_116_ fw	TTTGGGCTAACAGGAGGCTAGC ATATGAAAGACAGTATTACCAGC	pCEP_Xbud _01497_KM		GameXPeptide	this study
	DAS_117_rv	TCTGCAGAGCTCGAGCATGCAC ATGCGTCAGTAACGTGGTATG				
	DAS_118_V P_ rv	GCCGTAGGCATTCAATAACTG				

Table S5. NPs mentioned in this work including derivatives for some NPs. Structures are shown in Fig. 4B.

**Name**	**Compound**	**Detected** ***m/z*** $[M+H]^+$	**Reference**
GameXPeptide A (GXPA)	**1a**	586.4	
GameXPeptide B (GXPB)	**1b**	600.4	[11]
GameXPeptide C (GXPC)	**1c**	552.4	
Rhabdopeptide (RXP-599)	**2a**	599.2	
Rhabdopeptide (RXP-560)	**2b**	560.2	[12]
Rhabdopeptide (RXP-712)	**2c**	712.4	
Fabclavine – Ia	**3a**	1356.9	
Fabclavine – Ib	**3b**	1346.9	
Fabclavine – IIa	**3c**	1312.9	[13]
Fabclavine – IIb	**3d**	1302.9	
Pyrrolizixenamide	**4**	251.1	[14]
Xenobactin	**5**	756.4	[15]
Fabclavine – IVa	**6a**	1177.8	
Fabclavine – IVb	**6b**	1179.7	[13]
Fabclavine – IIIc	**6c**	1187.8	
Fabclavine – IIId	**6d**	1189.7	
PAX-peptide	**7**	1050.8	[16]
Xenocoumacin 2 (XCN2)	**8**	407.2	[17]
Xenorhabdin-299	**9**	299.1	[9]
Phenylethylamide-240	**10**	240.3	[10]
Rhabduscin	**11**	333.3	[18]

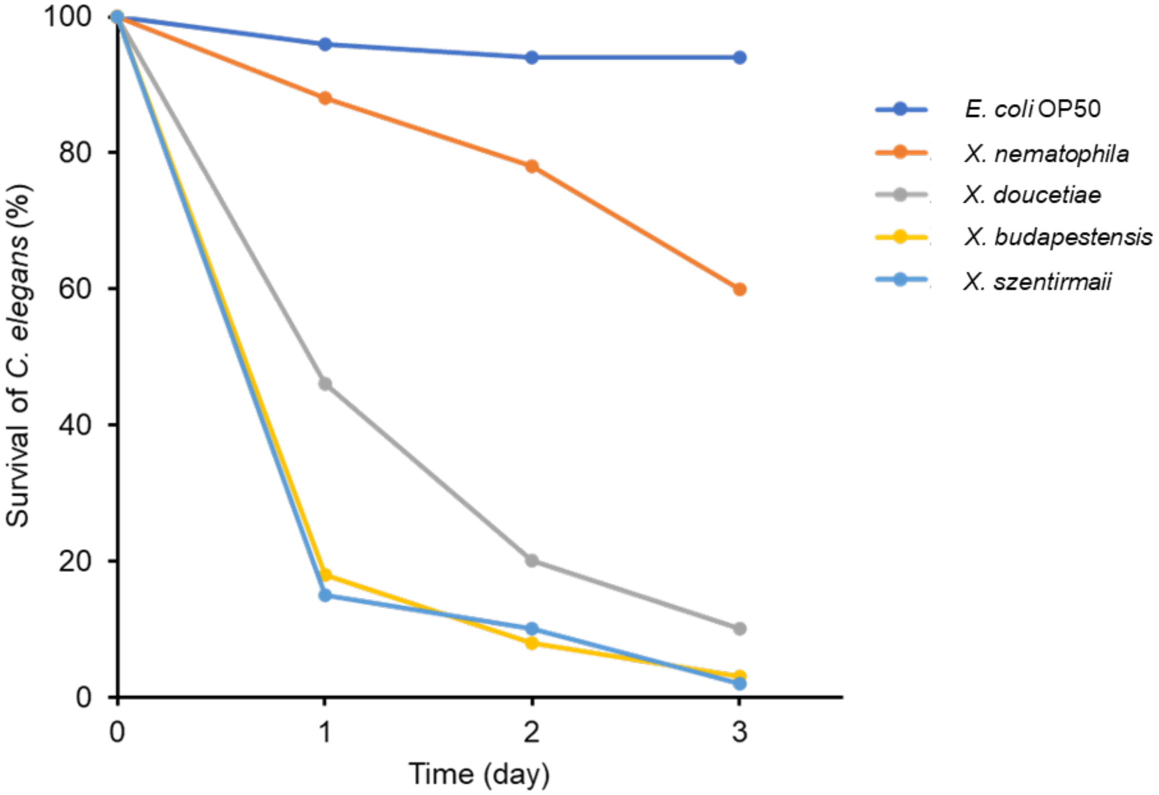

Figure S1. **Nematicidal activity of cell-free culture supernatant of *Xenorhabdus* WT strains.** Cell-free culture supernatant of *X. szentirmaii*, *X. budapestensis*, *X. doucetiae, X. nematophila* and *E. coli* OP50 (control). The cell-free culture supernatant of *E. coli* OP50 was used as a negative control. *X. szentirmaii*, *X. budapestensis* and *X. doucetiae* showed strong nematicidal activity compared to *X. nematophila* during three days of the experiment.

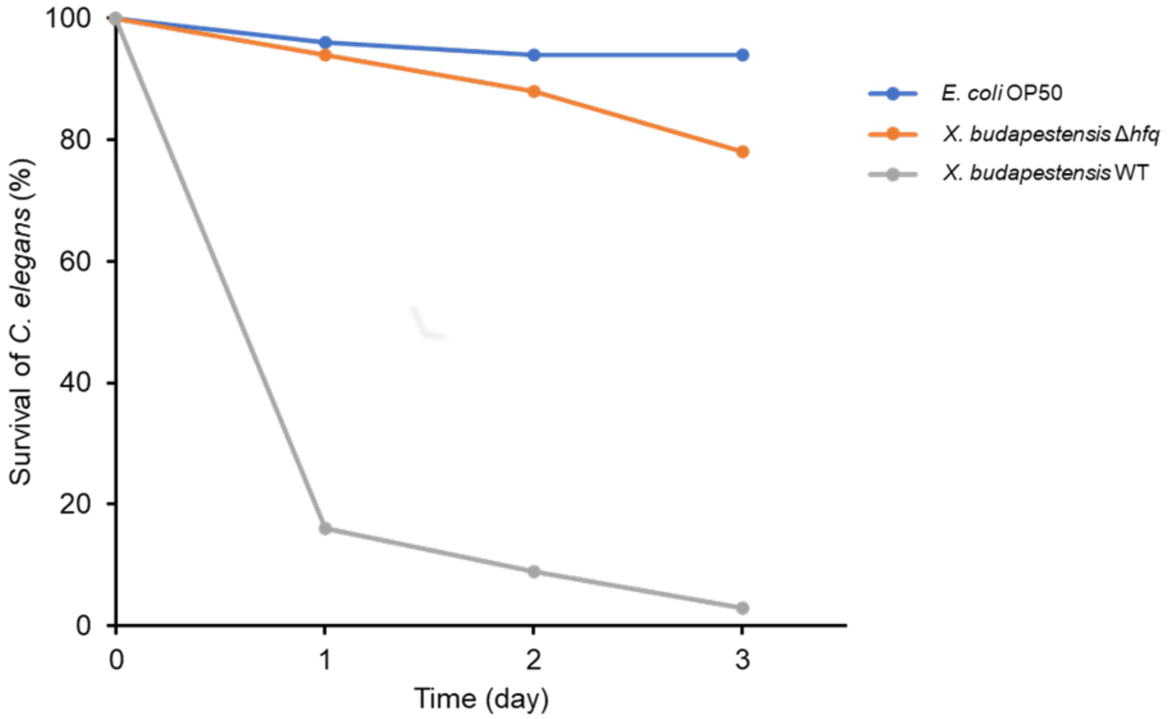

Figure S2. Nematicidal activity of cell-free culture supernatant of *X. budapestensis* WT compared to its Δ*hfq* strain. Cell-free culture supernatant of *X. budapestensis* WT showing strong nematicidal activity against *C. elegans* (L4) while cell-free culture supernatant of its Δ*hfq* strain is less potent like the that of the *E. coli* OP50 which was used as a control during three days of the experiment. Absence of nematicidal properties of the *X. budapestensis* WT is related to deletion of the *hfq* gene.

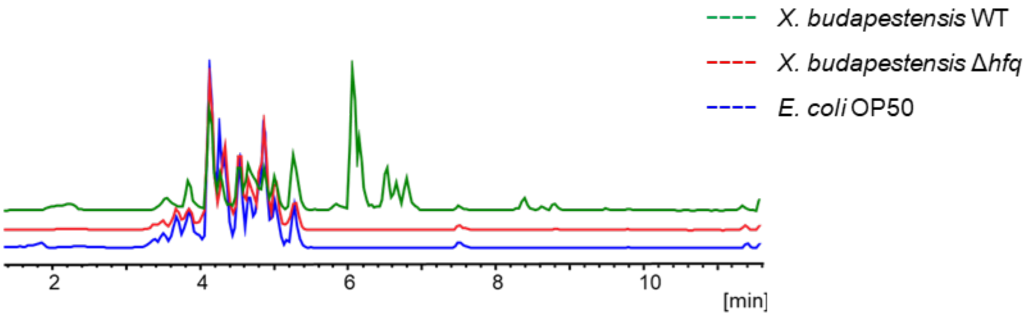

Figure S3. HPLC-MS data analysis for WT of *Xenorhabdus* and *E. coli* OP50. HPLC-MS data analysis of culture supernatant of WT of *X. budapestensis* (nematicidal *Xenorhabdus* bacteria), *X. budapestensis* Δ*hfq* (non-nematicidal strain, control), *E. coli* OP50 (non-nematicidal which is food of *C. elegans*, control). Profile of their Base Peak Chromatograms (BPC) agrees with their nematicidal activity. Nematicidal WT strain of *X. budapestensis* showed strong signals of natural products compared to the non-nematicidal strains.

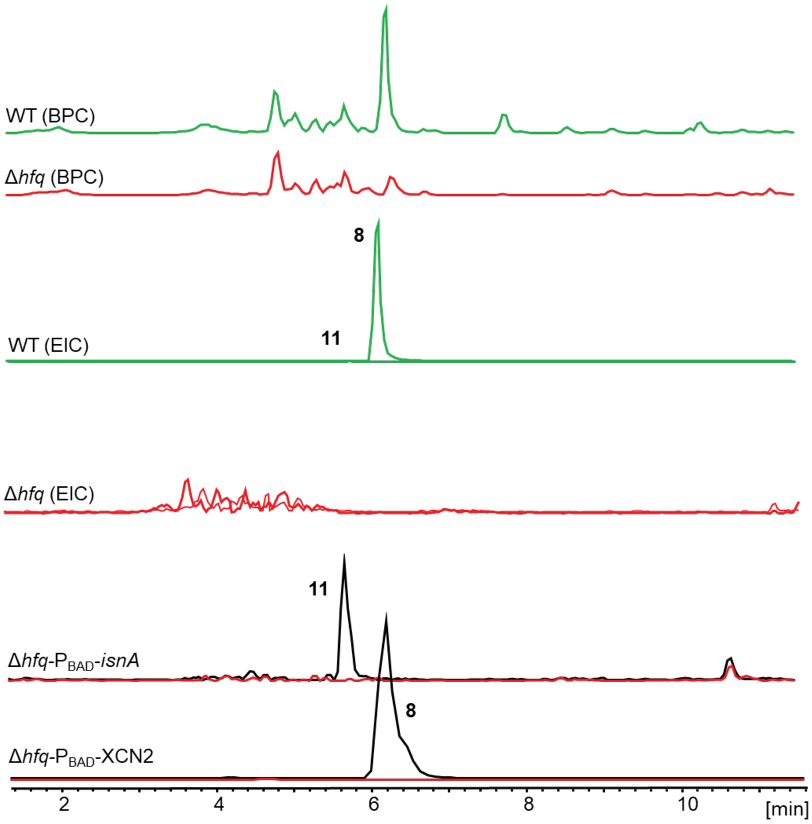

Figure S4. Exclusive production of natural products using promoter exchange. HPLC-MS data analysis of WT and Δ*hfq* mutant of *X. doucetiae* and NPs detected after a promoter exchange in the Δ*hfq* mutant. Extracted Ion Chromatograms (EICs) of xenocoumacin (**8**) and rhabduscin (**11**) are indicated to show their exclusive production.

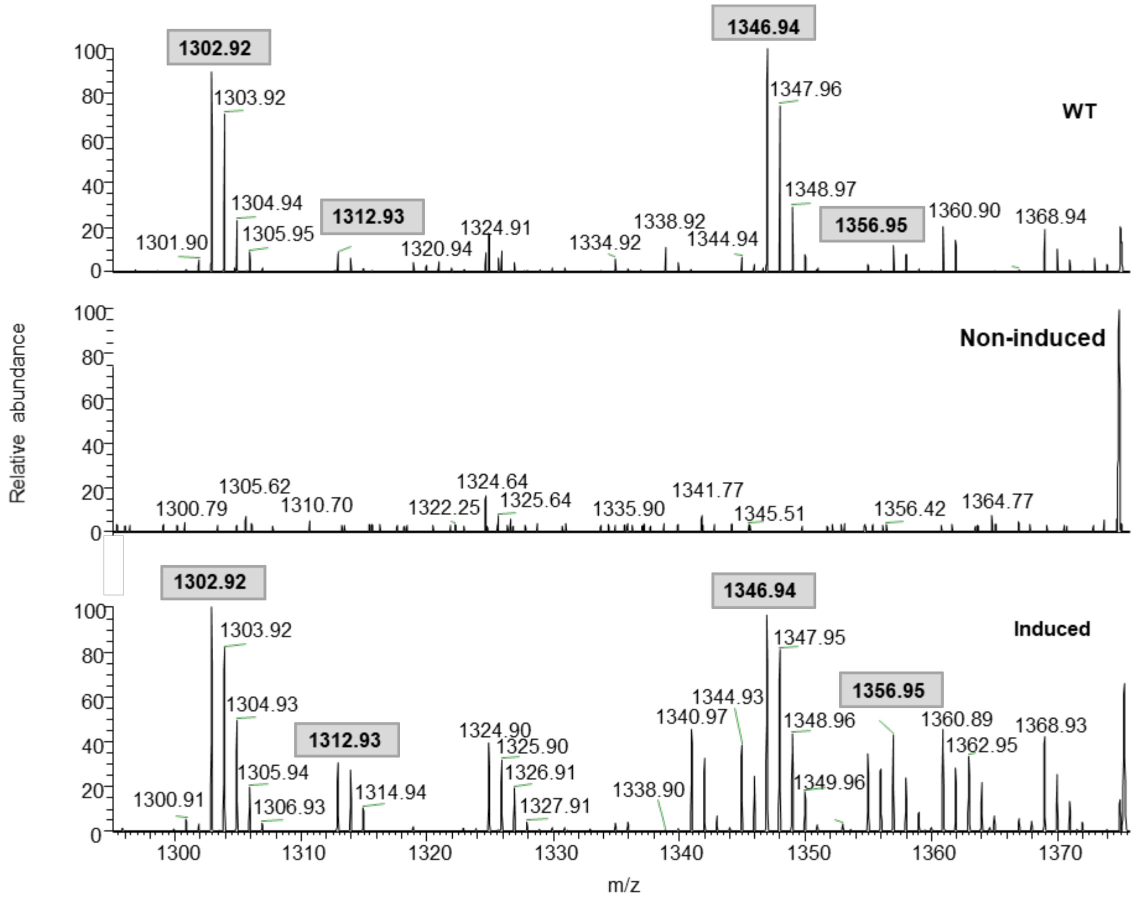

Figure S5. MALDI-MS spectra of fabclavine in *X. budapestensis* WT and its promoter exchange mutant. MALDI-MS analysis showing spectra of fabclavine derivatives in production culture of *X. budapestensis* WT and in the induced strain of *X. budapestensis*_Δ*hfq*_P_{BAD}*fclC.* Signals highlighted in bold are fabclavine derivatives (*m/z* $[M+H]^+$ = 1356.9, *m/z* $[M+H]^+$ = 1346.9, *m/z* $[M+H]^+$ = 1312.9; *m/z* $[M+H]^+$ = 1302.9) which were identified in *X. budapestensis*.[13] No production of fabclavine derivatives was detected in the non-induced strain of *X. budapestensis*_Δ*hfq*_P_{BAD}*fclC.*

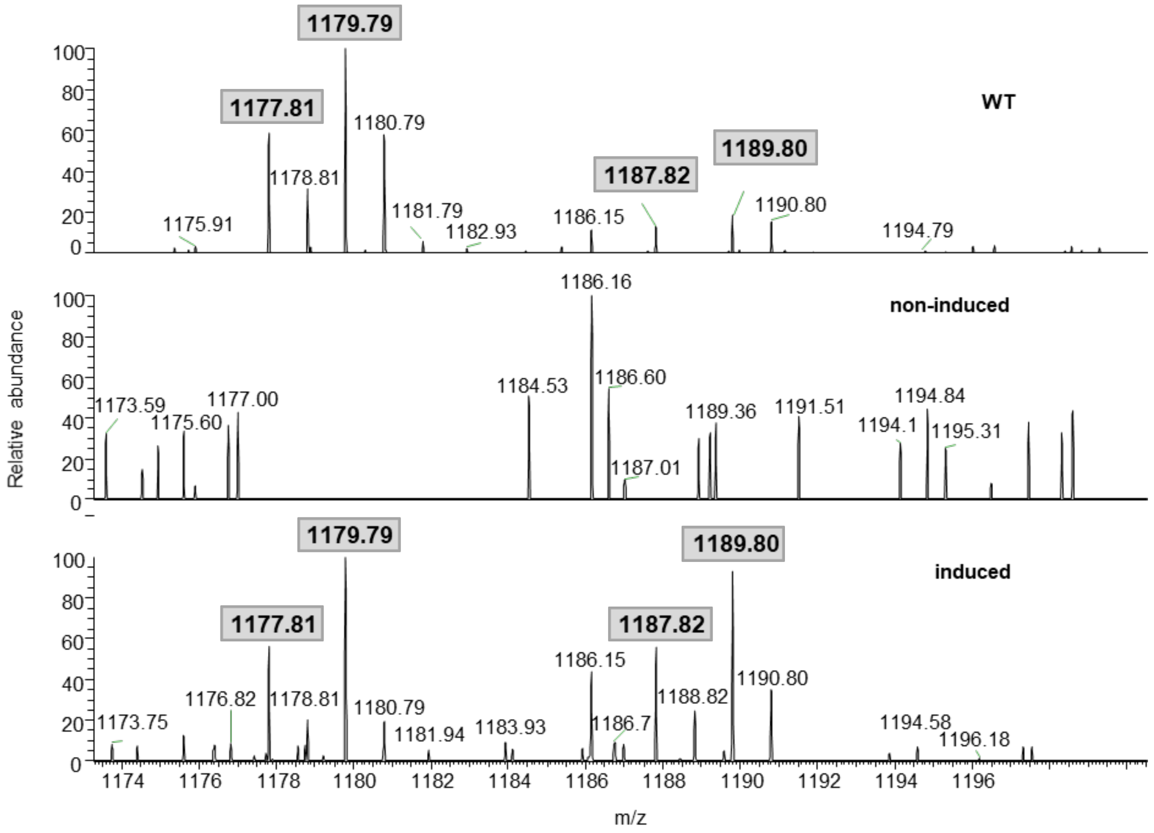

Figure S6. MALDI-MS spectra of fabclavine in *X. szentirmaii* WT and its promoter exchange mutant. MALDI-MS data analysis showing spectra of fabclavines in liquid culture of WT and Δ*hfq*_P_{BAD}*fclC* mutant strain of *X. szentirmaii*. Production of fabclavines was detected in liquid culture of WT and in an induced strain of *X. szentirmaii*_Δ*hfq*_P_{BAD}*fclC.* Signals highlighted in bold are fabclavine derivatives (*m/z* $[M+H]^+$ = 1177.8, *m/z* $[M+H]^+$ = 1179.7, *m/z* $[M+H]^+$ = 1187.8; *m/z* $[M+H]^+$ = 1189.8), which were identified in *X. szentirmaii*.[13] No production of fabclavine derivatives was detected in non-induced strain of *X. szentirmaii*_Δ*hfq*_P_{BAD}*fclC.*

References

(1) Zhang, J.; Holdorf, A. D.; Walhout, A. J. *C. elegans* and its bacterial diet as a model for systems-level understanding of host-microbiota interactions, *Curr. Opin. Biotechnol.* **2017**, *46,* 74–80.

(2) Babity, J. M.; Starr, T. V.; Rose, A. M. Tc1 transposition and mutator activity in a Bristol strain of *Caenorhabditis elegans*, *Mol. Gen. Genet.* **1990**, *222,* 65–70.

(3) Alford, D. V. *Pests of Ornamental Trees, Shrubs and Flowers;* CRC Press, 2012.

(4) Tailliez, P.; Pagès, S.; Ginibre, N.; Boemare, N. New insight into diversity in the genus *Xenorhabdus*, including the description of ten novel species, *Int. J. Syst. Evol.* **2006**, *56,* 2805–2818.

(5) Vivas, E. I.; Goodrich-Blair, H. *Xenorhabdus nematophilus* as a model for host-bacterium interactions: rpoS is necessary for mutualism with nematodes, *J. Bacteriol.* **2001**, *183,* 4687–4693.

(6) Gualtieri, M.; Ogier, J.-C.; Pagès, S.; Givaudan, A.; Gaudriault, S. Draft Genome Sequence and Annotation of the Entomopathogenic Bacterium *Xenorhabdus szentirmaii* Strain DSM16338, *Genome Announc.* **2014**, 2(2).

(7) Lengyel, K.; Lang, E.; Fodor, A.; Szállás, E.; Schumann, P.; Stackebrandt, E. Description of four novel species of *Xenorhabdus*, family Enterobacteriaceae: *Xenorhabdus budapestensis* sp. nov., *Xenorhabdus ehlersii* sp. nov., *Xenorhabdus innexi* sp. nov., and *Xenorhabdus szentirmaii* sp. nov, *Syst. Appl. Microbiol.* **2005**, *28,* 115–122.

(8) Bode, E.; He, Y.; Vo, T. D.; Schultz, R.; Kaiser, M.; Bode, H. B. Biosynthesis and function of simple amides in *Xenorhabdus doucetiae*, *Environ. Microbiol.* **2017**, *19,* 4564–4575.

(9) Bode, E.; Brachmann, A. O.; Kegler, C.; Simsek, R.; Dauth, C.; Zhou, Q.; Kaiser, M.; Klemmt, P.; Bode, H. B. Simple "on-demand" production of bioactive natural products, *Chembiochem.* **2015**, *16,* 1115–1119.

(10) Bode, E.; Heinrich, A. K.; Hirschmann, M.; Abebew, D.; Shi, Y.-N.; Vo, T. D.; Wesche, F.; Shi, Y.-M.; Grün, P.; Simonyi, S.; Keller, N.; Engel, Y.; Wenski, S.; Bennet, R.; Beyer, S.; Bischoff, I.; Buaya, A.; Brandt, S.; Cakmak, I.; Çimen, H.; Eckstein, S.; Frank, D.; Fürst, R.; Gand, M.; Geisslinger, G.; Hazir, S.; Henke, M.; Heermann, R.; Lecaudey, V.; Schäfer, W.; Schiffmann, S.; Schüffler, A.; Schwenk, R.; Skaljac, M.; Thines, E.; Thines, M.; Ulshöfer, T.; Vilcinskas, A.; Wichelhaus, T. A.; Bode, H. B. Promoter Activation in Δ*hfq* Mutants as an Efficient

Tool for Specialized Metabolite Production Enabling Direct Bioactivity Testing, *Angew. Chem. Int. Ed.* **2019**, *58,* 18957–18963.

(11) Bode, H. B.; Reimer, D.; Fuchs, S. W.; Kirchner, F.; Dauth, C.; Kegler, C.; Lorenzen, W.; Brachmann, A. O.; Grün, P. Determination of the absolute configuration of peptide natural products by using stable isotope labeling and mass spectrometry, *Chemistry.* **2012**, *18,* 2342–2348.

(12) Cai, X.; Nowak, S.; Wesche, F.; Bischoff, I.; Kaiser, M.; Fürst, R.; Bode, H. B. Entomopathogenic bacteria use multiple mechanisms for bioactive peptide library design, *Nat. Chem.* **2017**, *9,* 379–386.

(13) Fuchs, S. W.; Grundmann, F.; Kurz, M.; Kaiser, M.; Bode, H. B. Fabclavines: bioactive peptide-polyketide-polyamino hybrids from *Xenorhabdus*, *Chembiochem.* **2014**, *15,* 512–516.

(14) Schimming, O.; Challinor, V. L.; Tobias, N. J.; Adihou, H.; Grün, P.; Pöschel, L.; Richter, C.; Schwalbe, H.; Bode, H. B. Structure, Biosynthesis, and Occurrence of Bacterial Pyrrolizidine Alkaloids, *Angew. Chem. Int. Ed.* **2015**, *54,* 12702–12705.

(15) Grundmann, F.; Kaiser, M.; Kurz, M.; Schiell, M.; Batzer, A.; Bode, H. B. Structure determination of the bioactive depsipeptide xenobactin from *Xenorhabdus* sp. PB30.3, *RSC Adv.* **2013**, *3,* 22072.

(16) Fuchs, S. W.; Proschak, A.; Jaskolla, T. W.; Karas, M.; Bode, H. B. Structure elucidation and biosynthesis of lysine-rich cyclic peptides in *Xenorhabdus nematophila*, *Org. Biomol. Chem.* **2011**, *9,* 3130–3132.

(17) Reimer, D.; Luxenburger, E.; Brachmann, A. O.; Bode, H. B. A new type of pyrrolidine biosynthesis is involved in the late steps of xenocoumacin production in *Xenorhabdus nematophila*, *Chembiochem.* **2009**, *10,* 1997–2001.

(18) Crawford, J. M.; Portmann, C.; Zhang, X.; Roeffaers, M. B. J.; Clardy, J. Small molecule perimeter defense in entomopathogenic bacteria, *Proc. Natl. Acad. Sci. U.S.A.* **2012**, *109,* 10821–10826.
